# Genomic insights into *Aspergillus sydowii* 29R-4-F02: unraveling adaptive mechanisms in subseafloor coal-bearing sediment environments

**DOI:** 10.3389/fmicb.2023.1216714

**Published:** 2023-06-29

**Authors:** Jun-Peng Jiang, Xuan Liu, Yi-Fan Liao, Jun Shan, Yu-Ping Zhu, Chang-Hong Liu

**Affiliations:** ^1^State Key Laboratory of Pharmaceutical Biotechnology, Nanjing University, Nanjing, China; ^2^State Key Laboratory of Soil and Sustainable Agriculture, Institute of Soil Science, Chinese Academy of Sciences, Nanjing, China

**Keywords:** *Aspergillus sydowii*, subseafloor sediments, fungi, CAZymes, adaptive evolution

## Abstract

**Introduction:**

*Aspergillussydowii* is an important filamentous fungus that inhabits diverse environments. However, investigations on the biology and genetics of *A. sydowii* in subseafloor sediments remain limited.

**Methods:**

Here, we performed *de novo* sequencing and assembly of the *A. sydowii* 29R-4-F02 genome, an isolate obtained from approximately 2.4 km deep, 20-million-year-old coal-bearing sediments beneath the seafloor by employing the Nanopore sequencing platform.

**Results and Discussion:**

The generated genome was 37.19 Mb with GC content of 50.05%. The final assembly consisted of 11 contigs with N_50_ of 4.6 Mb, encoding 12,488 putative genes. Notably, the subseafloor strain 29R-4-F02 showed a higher number of carbohydrate-active enzymes (CAZymes) and distinct genes related to vesicular fusion and autophagy compared to the terrestrial strain CBS593.65. Furthermore, 257 positively selected genes, including those involved in DNA repair and CAZymes were identified in subseafloor strain 29R-4-F02. These findings suggest that *A. sydowii* possesses a unique genetic repertoire enabling its survival in the extreme subseafloor environments over tens of millions of years.

## Introduction

Ocean Drilling Program revealed a new ecosystem composed of microorganisms in subseafloor sediments (D’Hondt et al., 2004; Schrenk et al., 2010; Lomstein et al., 2012), which vertically extends for ~2.5 km below the seafloor (Selbmann et al., 2013; Inagaki et al., 2015). This deep subseafloor biosphere harbors organisms from three major domains of life: Archaea (Orsi et al., 2013), Bacteria (Schippers et al., 2005), and Eukarya (Borgonie et al., 2015). Fungi, as the dominant component of eukaryotes, also widely exist in the subseafloor sediments (Rédou et al., 2015; Nagano et al., 2016; Liu et al., 2017). Most subseafloor fungi were affiliated with the phyla Ascomycota, Basidiomycota and Chytridiomycota (Nagano and Nagahama, 2012). Phylogenetic analysis indicated that subseafloor fungi were closely related to the isolates from terrestrial, freshwater and marine environments (Liu et al., 2017; Quemener et al., 2020). Various culture-dependent and culture-independent investigations show that fungi are active and well-adapted to subseafloor environments (Rédou et al., 2015; Liu et al., 2017). Some fungi, such as *Aquamyces* sp. and *Orbilia* sp., can live in a saprophytic mode and maintain their life in anaerobic habitats by degrading organic substances (Ortega-Arbulu et al., 2019). Our previous studies have shown that *Schizophyllum commune* isolated from ~2 km coal-bearing sediment below seafloor has a variety of anaerobic survival strategies, including improving the metabolic efficiency of ethanol and amino acids, increasing the number of mitochondria in cells, and forming autophagosomes, but it cannot complete sexual propagation (Zain Ul Arifeen et al., 2021a,b). Genome analysis of *S. commune* revealed significant expansion of FunK1 protein kinase, the NmrA family, and transposon, but significant reduction of nucleotide diversity, substitution rates, and homologous recombination, reflecting the unique genetic evolution of fungi to maintain their long-term life in subseafloor environments (Liu et al., 2022). However, due to the limited number of pure culture strains of subseafloor fungi, the genomes of only a few fungi have been sequenced, and there is still a lack of understanding of the genetic characteristics of the fungi in deep biosphere.

Aspergillus is one of the best-studied filamentous fungi, mainly because some species of this genus are widely used in the fields of medicine (e.g., *A. fumigatus*, *A. terreus*), food spoilage (e.g., *A. flavus*, *A. parasiticus*, and *A. hancockii*), and industry (e.g., *A. niger*, *A. aculeatus*, and *A. oryzae*; de Vries et al., 2017; Pitt et al., 2017). *A. sydowii*, in particular, is distributed throughout the world and can survive as saprophytes (Raper and Fennell, 1965; Geiser et al., 1998; Klich, 2002). Our previous study found that this fungus is also one of the dominant fungi in ~2 km sediments below the seafloor (Liu et al., 2017). Some strains of *A. sydowii* isolated from marine ecosystems can cause an epizootic infection of sea fan corals (Kim and Harvell, 2004; Hayashi et al., 2016), while others can produce a series of secondary metabolites with novel structure and bioactivities (Wang et al., 2019; Niu et al., 2020), and degrade the refractory substances such as xylan (Brandt et al., 2020), lignocellulose (Cong et al., 2017), and waste engine oil (Kumar et al., 2021). Given the wide distribution and important application value of *A. sydowii*, five strains of this species have been sequenced, assembled and annotated (de Vries et al., 2017; Brandt et al., 2021; Kumar et al., 2021; Jing et al., 2022), of which strain CBS593.65 had been fully studied. Of the sequenced strains, AS31, AS42, Fsh102, CBS593.65, and BOBA1 were isolated from terrestrial land and deep sea, respectively. However, the genome of any *A. sydowii* strains derived from subseafloor sediments is not yet available.

In order to acquire insight into the survival and environmental adaptation mechanism of subseafloor fungi, we performed the *de novo* whole genome assembly and comprehensive analysis of *A. sydowii* 29R-4-F02 buried in 2,405 m coal-bearing sediment below the seafloor. A large number of genes were identified to be involved in the secondary metabolism and carbohydrate metabolism. The carbohydrate-active enzymes (CAZymes) repertoire of *A. sydowii* 29R-4-F02 was identified and compared with that of other fungi. Moreover, genes or gene families were investigated through comparative genomes to reveal the special genetic evolution process of the strain.

## Materials and methods

### Fungal strain and culture conditions

*A. sydowii* 29R-4-F02 (CCTCC AF 2022080) strain was isolated from ~2.4 km coal-bearing sediments below the seafloor at drilling Site C0020 (41°10′35″N, 142°12′01″E) off the Shimokita Peninsula, Japan (Liu et al., 2017) and maintained aerobically on potato dextrose agar (PDA, 200 g/L potato, 20 g/L glucose, and 15 g/L agar) slants at 4°C. Details of the habitat and culture conditions of *A. sydowii* 29R-4-F02 have been described previously (Liu et al., 2017). For DNA isolation, mycelium of *A. sydowii* 29R-4-F02 was grown in 250 ml conical flask containing 100 ml PD (200 g/L potato and 20 g/L glucose) and incubated at 30°C and 200 rpm for 2–3 days.

### Genome sequencing and assembly

DNA extraction was carried out by HP fungal DNA mini kit (Omega Bio-Tek, GA, USA) according to the manufacturer’s instructions. The DNA concentration (>50 ng/μl), quality and integrity were determined by using a Qubit Flurometer (Invitrogen, USA) and a NanoDrop Spectrophotometer (Thermo Scientific, USA). Genome sequencing was performed using the Nanopore sequencing platform at the Beijing Novogene Bioinformatics Technology Co., Ltd. After filtering out low quality reads (less than 500 bp) by Nanoplot v 1.41.0 software, a total of 441,200 bases reads was retained (De Coster et al., 2018). The clean reads were mapped with each other to find the overlap between sequences by Minimap2 v 2.26 software (Li, 2018), and then assembled by Miniasm software to constructed scaffolds (Li, 2016). Finally, the Racon v 1.5.0 software was used to constructed consensus sequence and correct the assembly results (Vaser et al., 2017). BUSCO v 5.4.7 was used to assess the completeness of gene regions using the dataset fungi_odb9 (Waterhouse et al., 2017). Quast v 5.1.0 software was further used to evaluate the assembly quality of *A. sydowii* 29R-4-F02 genome with the default parameters (Gurevich et al., 2013). Reference-based assembly of the *A. sydowii* 29R-4-F02 mitochondrial genome was conducted using the Rebaler v 0.2.0 software (Baeza and García-De León, 2022). We executed Rebaler using *A. nidulans* mitochondrial genome as reference genome. Also, we ran Rebaler with the option “circular = true” indicating that the reference genome was circular so that Rebaler “rotated” contigs between polishing rounds to ensure improved accuracy of the final assembled mitochondrial genome.

### Genome annotation

After obtaining the whole-genome data of *A. sydowii* 29R-4-F02, we used two strategies to annotated genes: (i) Augustus v 2.7 was used for *de novo* prediction (Stanke et al., 2008); (ii) Homologous species was predicted in Genewise v 2.4.1 using the reference gene models of *A. sydowii* CBS593.65 (Birney et al., 2004). Gene models from these two approaches were combined using the EVidenceModeler v 2.1.0 and updated by PASA v2.4.1 (Haas et al., 2008). Dispersed repeat sequences (DRs) and tandem repeat sequences (TRs) were identified by RepeatMasker v open-4.0.5 (Saha et al., 2008) and TandemRepeat v 4.07b Finder (TRF), respectively (Benson, 1999). The tRNA and rRNA were predicted by tRNAscan-SE v 1.3.1 (Chan et al., 2021) and rRNAmmer v 1.2 (Lagesen et al., 2007). The sRNA, snRNA and miRNA were first performed Rfam database v 31.0 (Gardner et al., 2009) comparison annotation, and then use the cmsearch program (v 1.1rc4; default parameter) to determine the final sRNA, snRNA and miRNA.

Gene functions were annotated based on Gene Ontology (GO, v 20171011), Kyoto Encyclopedia of Genes and Genomes (KEGG, v 20181107), Eukaryotic Clusters of Orthologous Groups (KOG, v 201711), Non-redundant protein database (Nr, v 201703), PFAM databases (v 31.0), SwissProt (v 20180716), and Carbohydrate-Active enZYmes Database (v 20181107; Wang et al., 2021; Liu et al., 2022). The secreted proteins were predicted by the SingalP 4.1 and TMHMM 2.0c (Petersen et al., 2011) signal database. Meanwhile, fungal secondary metabolite pathways were predicted using the online tool AntiSMASH v 4.0.2 (Medema et al., 2011). The cytochrome P450 gene family were predicted using Cytochrome P450 Database (Fischer et al., 2007).

### Phylogenetic and synteny analyses

A total of 21 fungal species (i.e., 22 strains) of *Aspergillus* with whole genome sequence, including *A. sydowii* (two strains 29R-4-F02 and CBS593.65), *A. aculeatus*, *A. brasiliensis*, *A. carbonarius*, *A. clavatus*, *A. fischeri*, *A. flavus*, *A. fumigatus*, *A. glaucus*, *A*. *homomorphus*, *A. ibericus*, *A*. *luchuensis*, *A. nidulans*, *A. niger*, *A. oryzae*, *A. phoenicis*, *A. terreus*, *A*. *tubingensis*, *A. versicolor*, *A*. *wentii*, and *A. zonatus* were used to perform gene family analysis. Except *A*. *sydowii* 29R-4-F02, protein sequences of these species were downloaded from NCBI and JGI databases, and analyzed by OrthoFinder with default parameters to find orthologous genes families (Emms and Kelly, 2015).

The single copy orthologous genes of the 22 analyzed strains were aligned using MAFFT and create a super-gene for each species (Katoh and Standley, 2013). After Gblocks alignment optimization (Castresana, 2000), the conserved blocks of super-genes were used for phylogenetic tree construction using RAxML (Stamatakis, 2014). The final phylogenetic tree was visualized and edited in iTOL9 (Letunic and Bork, 2016).

In addition, synteny analysis of strains 29R-4-F02 and CBS593.65 was performed using MUMmer sequence alignment package (Delcher et al., 2002). Conserved syntenic blocks between the two strains were identified using the MCScanX package with default parameters based on a minimum requirement of five collinear orthologous genes (Wang et al., 2012). Gene Ontology (GO) enrichment was analyzed by Cytoscape software.

### Identification and classification of CAZymes families

For comparative analysis of the CAZymes, HmmScan in the HMMER v 3.1b2 package (Eddy, 1998) was used to search protein sequences of *A. sydowii* 29R-4-F02 against the family-specific HMM profiles of CAZymes from dbCAN database v 9 (Yin et al., 2012) to detect putative CAZymes. The identified CAZymes were then classified into glycoside hydrolases (GHs), glycosyltransferases (GTs), polysaccharide lyases (PLs), carbohydrate esterases (CEs), auxiliary activities (AAs), and carbohydrate-binding modules (CBMs) base on the CAZy database (Lombard et al., 2014).

### Positively selected genes

The positive selected genes (PSGs) between *A. sydowii* 29R-4-F02 and *A. sydowii* CBS593.65 were detected using the KaKs_Calculator v 2.0 software with the default parameters (Zhang et al., 2006).

## Results

### Genome sequencing and assembly

**Figure 1:**
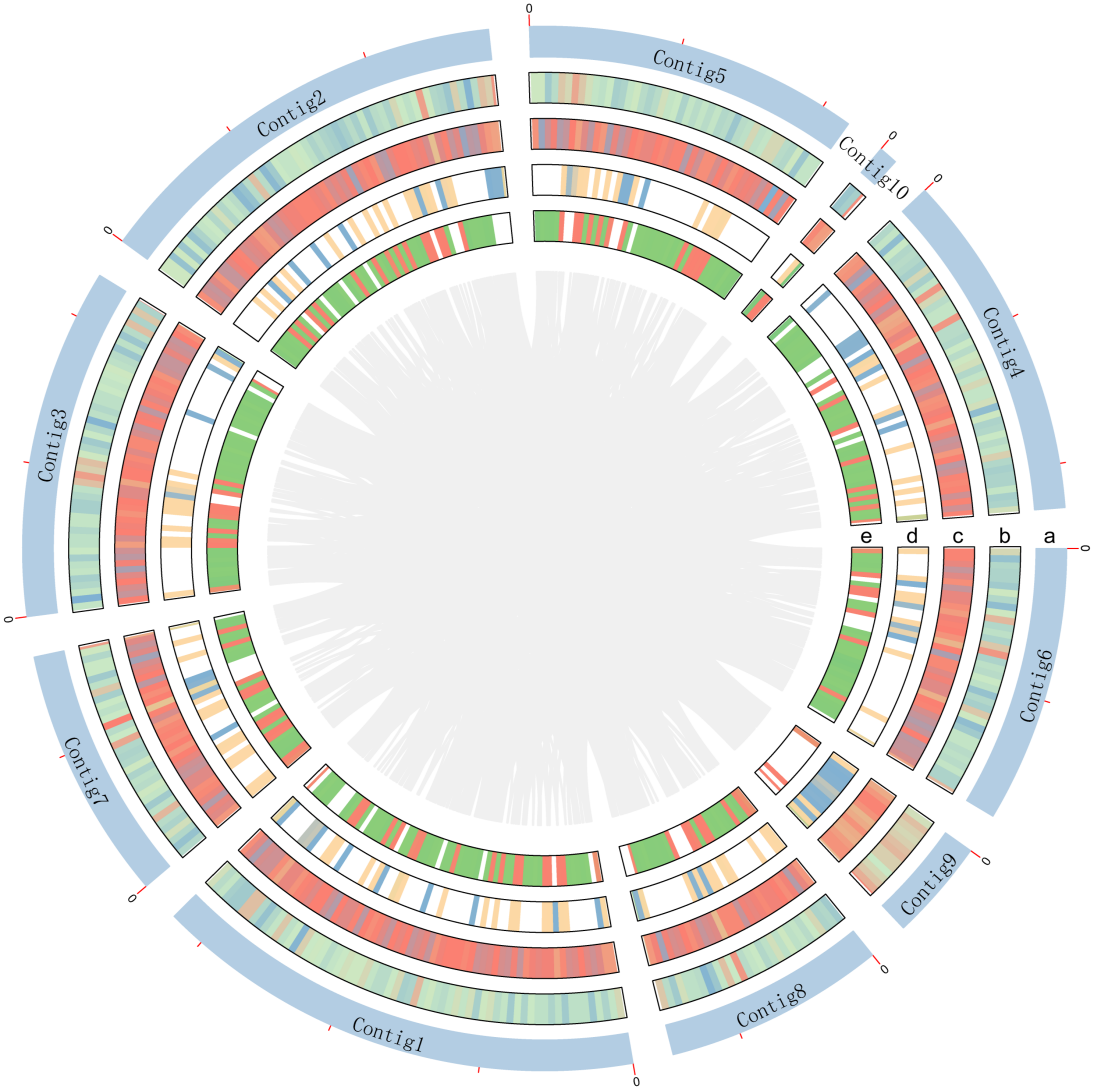
Schematic representation of genomic characteristics of *A. sydowii* 29R-4-F02. Circle a represents contigs, but excluding contig11 without annotation gene. Circle b and c are CDS on the positive and negative chains. Circle d and e are gene density on the positive and negative chains.

The genome of *A*. *sydowii* 29R-4-F02 was sequenced using Nanopore sequencing platform. After filtering out low-quality reads, over 3.11 Gb reads (~83.62×) were assembled into 11 contigs with a contig N50 of 4.6 Mb, and the GC content of 50.05% (Figure 1; Table 1), resulting in a total assembly size of 37.19 Mb that was consistent with the estimated genome size of 29.00–39.00 Mb and GC content of 48.00–53.00% of different *Aspergillus* isolates (de Vries et al., 2017; Kumar et al., 2021). The integrity of the gene region was evaluated using BUSCO, which showed high quality of sequence assembly, with 92.8% of single copies intact and only 3.7% missing. Quast analysis showed that the genomic quality of strain 29R-4-F02 was superior to that of strains Fsh102 and BOBA1 (Supplementary Figure 1), and comparable to that of strain CBS593.65 (de Vries et al., 2017). 86.10% of genes of strain 29R-4-F02 were collinear with those of strain CBS593.65 (Supplementary Figure 2). In addition, we assembled the mitochondrial genome of strain 29R-4-F02, which has a length of 26,185 bp and contains 13 genes, including 7 NADH–ubiquinone oxidoreductase genes, 3 cytochrome c oxidase genes, 1 cytochrome b gene, 1 ribosomal protein gene, and 1 ATP synthase gene, with a GC content of 23.67%. The size and number of genes in the mitochondrial genome of strain 29R-4-F02 are similar to those of other *Aspergillus* species, with genome sizes ranging from 27,817 to 77,649 bp and gene numbers ranging from 14 to 21 (Zhao et al., 2012; Takahashi et al., 2021; Jing et al., 2022).

**Table 1:** Genome assembly and annotation summary of *A. sydowii* strains.

Assembly feature	29R-4-F02	CBS593.65	Fsh102	BOBA1
Genome size (Mb)	37,191,710	34,381,026	35,409,723	38,795,664
Coverage (X)	83.6×	95.1×	88.65×	100.0×
Max Length (bp)	6,486,838	5,537,812	860,766	353,923
Number of Scaffold	11	97	474	2,582
Scaffold N50	4,575,881	2,288,531	260,502	31,598
Scaffold L50	4	5	40	330
GC content (%)	50.05	49.98	50.5	52.18
Gene number	12,488	13,717	10,761	18,932
Reference	In this study	de Vries et al. (2017)	Brandt et al. (2021)	Kumar et al. (2021)

### Genome and functional annotation

A total of 12,488 protein-coding genes were predicted with an average sequence length of 1,567 bp and a cumulative length of 19.6 Mb, accounting for 52.62% of the total genome sequence. The total length of repeat sequences was about 0.76 Mb, accounting for 2.06% of the genomic size. Among the repetitive elements, tandem and interspersed repeats account for 0.74 and 1.31%, respectively. Moreover, 160 tRNA genes, 35 sRNA genes, 15 snRNA genes, and 48 rRNA genes were predicted in the genome (Supplementary Table 1). Additionally, functional annotation of the predicted genes was performed based on the public database of Nr, SwissProt, KEGG, KOG, GO, and Pfam, protein databases (Supplementary Table 2).

**Figure 2:**
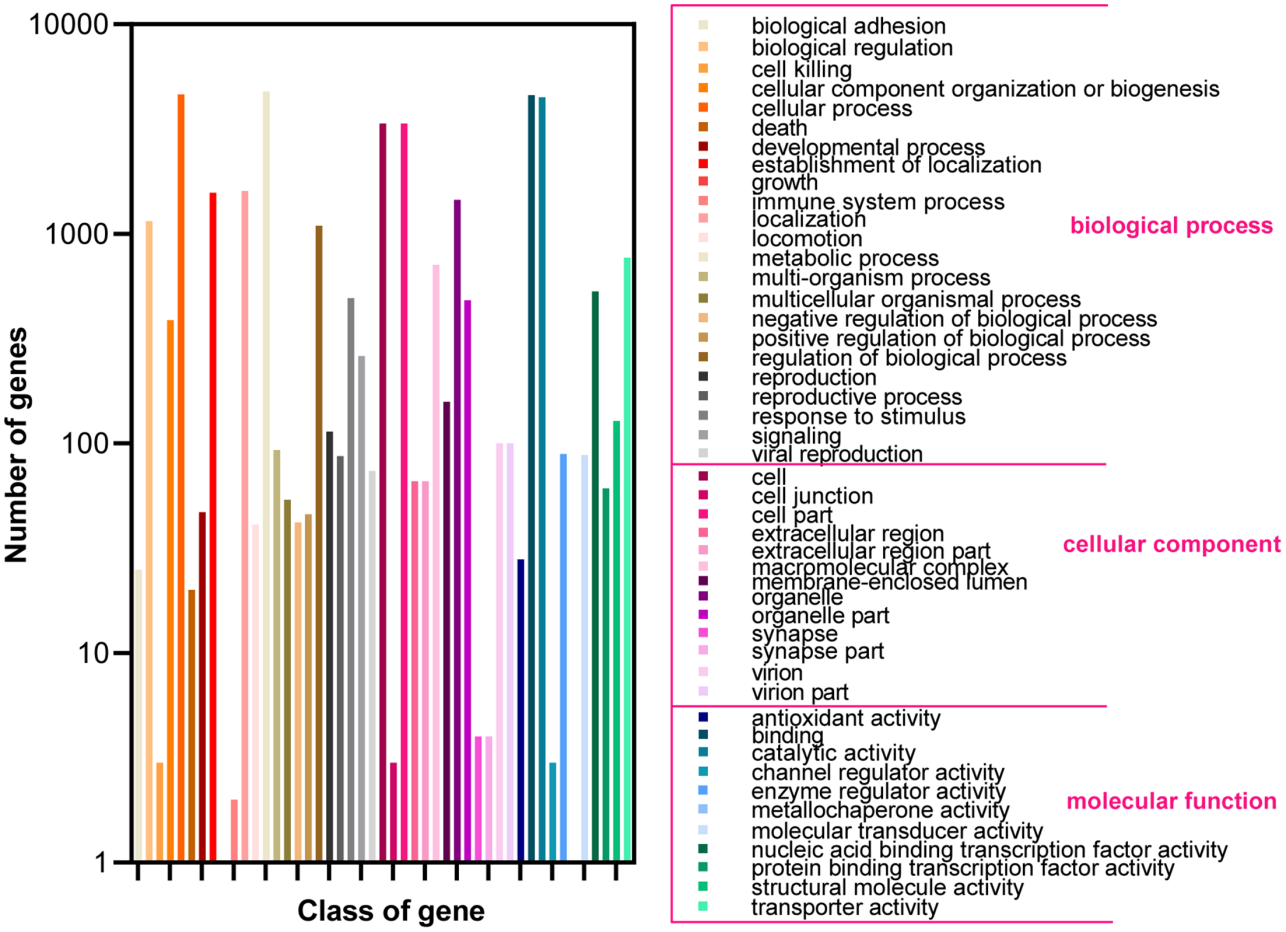
Functional annotation of *A. sydowii* 29R-4-F02 in Gene Ontology (GO) database.

**Figure 3:**
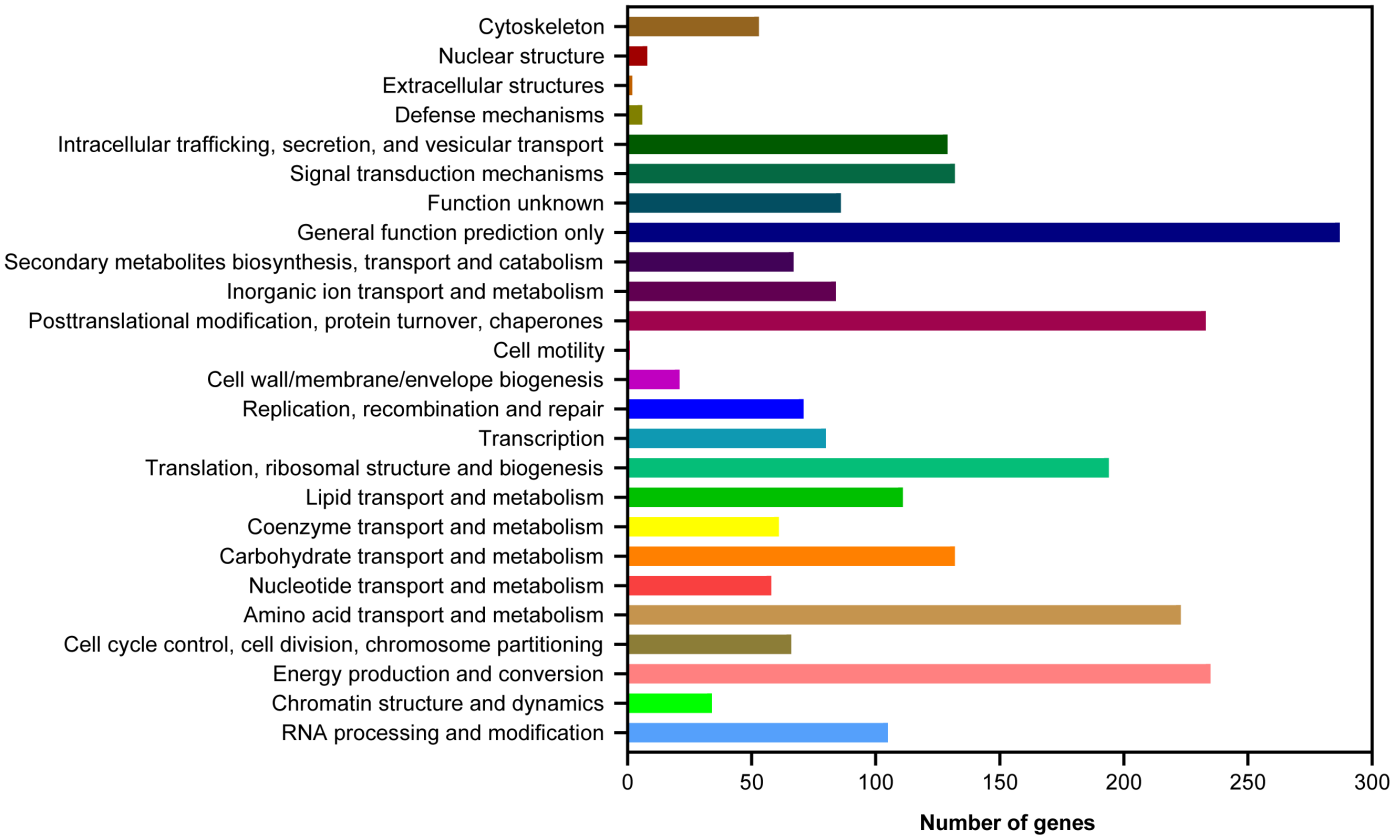
Clusters of Orthologous Groups of proteins (KOG) function classification of proteins in *A. sydowii* 29R-4-F02.

**Figure 4:**
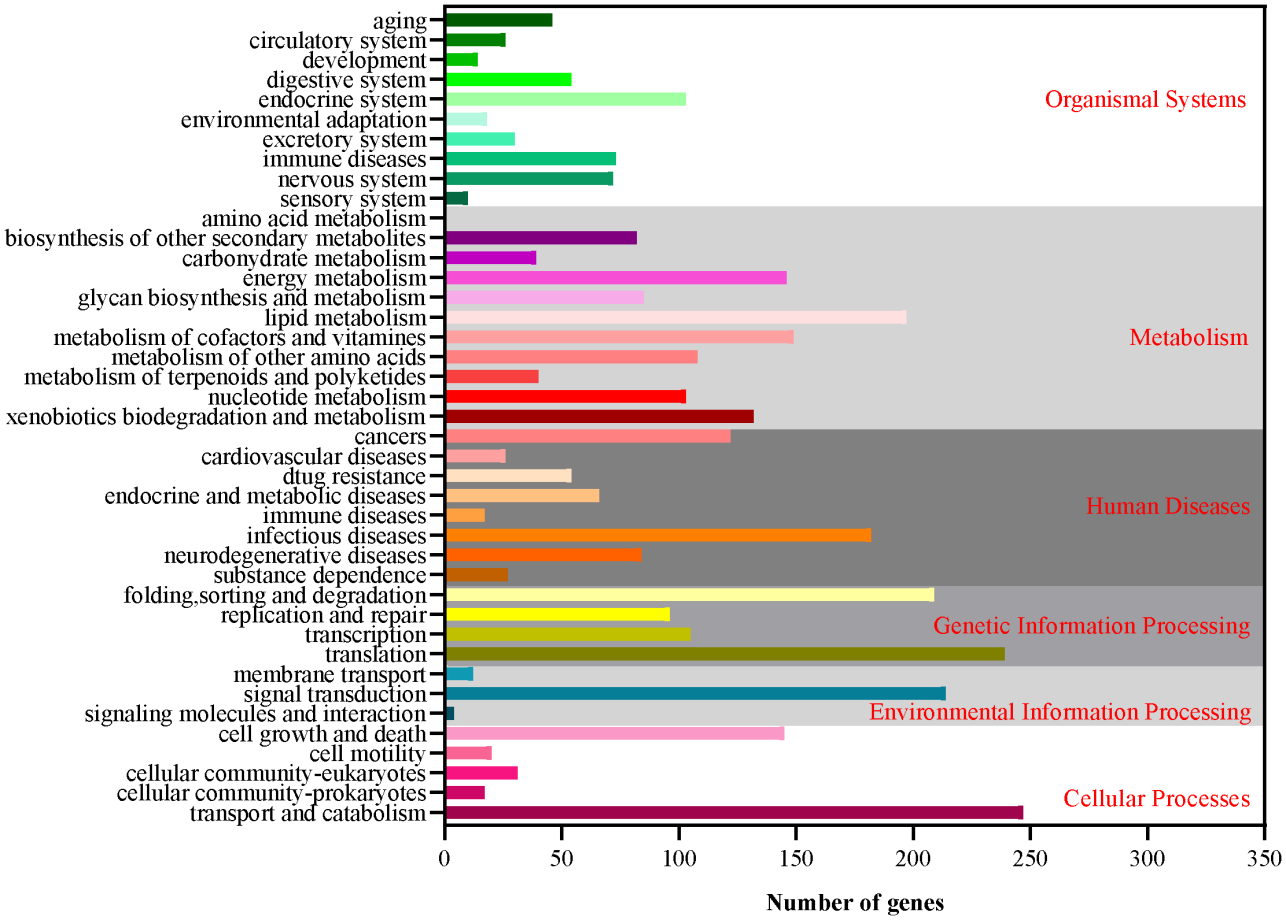
The Kyoto Encyclopedia of Genes and Genomes (KEGG) function annotation of *A. sydowii* 29R-4-F02.

Based on the GO database, 68.51% of protein-coding genes (8,555) were annotated into three categories: biological process, cellular components, and molecular function (Figure 2; Supplementary Table 2). For biological processes, genes involved in “metabolic process” (4,783) are the most abundant, followed by “cellular process” (4,637), “localization” (1,606), “establishment of localization” (1,570), “biological regulation” (1,150), and “regulation of biological process” (1,150). Among the genes involved in cellular components, 3,368, 3,368, 1,453, and 712 were associated with “cell,” “cell parts,” “organelle,” and “macromolecular complex,” respectively. Furthermore, for genes related to molecular function, 4,585, 4,487, 769, and 531 were involved in “binding,” “catalytic activity,” “transporter activity,” and “nucleic acid binding transcription factor activity,” respectively. These results revealed that the subseafloor *A. sydowii* 29R-4-F02 had abundant genes related to metabolic activities. Additionally, 2,237 protein-coding genes (17.91%) were annotated based on the KOG database (Figure 3; Supplementary Table 2). The majority of the genes were associated with “general function prediction only” (287), followed by “Energy production and conversion,” “post-translational modification, protein turnover, chaperones,” “Amino acid transport and metabolism,” and “Translation, ribosomal structure and biogenesis.” These findings indicated that *A. sydowii* 29R-4-F02 has a variety of protein metabolism and energy metabolism processes, which may help the fungus to better absorb and utilize nutrients from the subseafloor environment and maintain long-term survival. To further investigate the gene functions in *A. sydowii* 29R-4-F02, 10,893 (87.22%) protein-coding genes were assigned to their orthologs in the KEGG database. The KEGG function classification was shown in Figure 4 and Supplementary Table 2, including Cellular Processes (460), Environmental Information Processing (230), Genetic Information Processing (649), Human Diseases (578), Metabolism (1,756), and Organismal Systems (428). Consistent with the KOG annotation, metabolism and biosynthesis categories in KEGG were significantly annotated.

**Figure 5:**
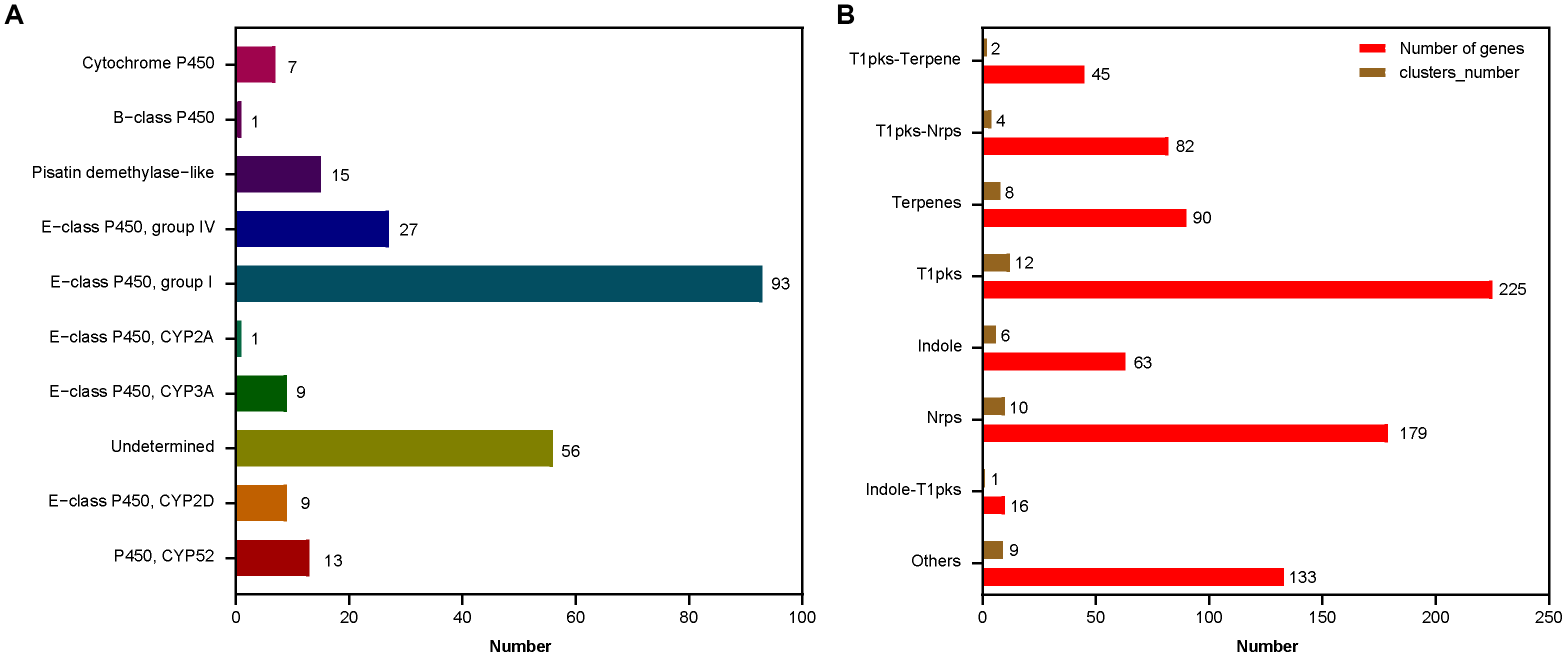
The function classification of the P450 genes **(A)** and the secondary metabolic gene clusters **(B)** in *A. sydowii* 29R-4-F02 genome. The horizontal axis represents the number of genes or gene clusters.

A total of 52 gene clusters involved in the secondary metabolism, 231 cytochrome P450 genes, and 920 secreted proteins were identified in *A. sydowii* 29R-4-F02 genome (Figure 5; Supplementary Table 3). Among these P450 genes (Figure 5A), 13 were classified as P450, CYP52 class, 9 as E − class P450, CYP2D class, 9 as E − class P450, CYP3A class, 1 as E − class P450, CYP2A class, 93 as E − class P450, group I class, 27 as E − class P450, group IV class, 15 as Pisatin demethylase−like class, 1 as B − class P450 class, 7 as Cytochrome P450 class, and 56 as undetermined class. The E-class P450, group I class encoded by the most genes are involved in the oxidation–reduction reactions, while 56 undetermined class were predicted to participate in various secondary metabolic processes, such as xenobiotic biodegradation and carbohydrate metabolism. Similarly, among the secondary metabolite gene clusters (Figure 5B), 12 were classified as T1pks, 10 as Nrps, 8 as Terpenes, 6 as Indole, 4 as T1pks-Nrps, 2 as T1pks-Terpene, 1 as Indole-T1pks, and 9 others. These clusters contain whole or partial genes from known clusters such as Indole-T1pks and T1pks-Terpene cluster from *Penicillium digitatum* (CBS130527; Wang et al., 2021), T1pks and Terpene cluster from *Calcarisporium* sp. (KF525; Kumar et al., 2018), Nrps cluster from *Aspergillus hancockii* (FRR3425; Pitt et al., 2017), T1pks-Nrps cluster from *Aspergillus westerdijkiae* (CBS112803; Han et al., 2016), and T1pks-Nrps cluster from *Pestalotiopsis* sp. (KF079; Kumar et al., 2018), suggesting that *A. sydowii* 29R-4-F02 is capable of synthesizing these metabolites. These genes play a crucial role in fungi adapting to extreme environments and maintaining population competitive advantages (Choi et al., 2010; Cresnar and Petric, 2011; Keller, 2019; Yurchenko et al., 2021).

### The CAZyme family

CAZymes play important roles in degrading cellulose, hemicellulose, pectin, and lignin polysaccharides and are responsible for the acquisition of nutrients for fungi (de Vries et al., 2017). In *A. sydowii* 29R-4-F02 genome, 670 CAZyme coding genes were identified, including 337 encoding genes for GHs, 78 encoding genes for AAs, 126 encoding genes for GTs, 39 encoding genes for CEs, 67 encoding genes for CBMs, and 23 encoding genes for PLs (Supplementary Table 4), which was much more than the genes in other species of *Aspergillus* (Table 2). Strain 29R-4-F02 also contains a large number of CAZyme coding genes related to the degradation of plant cell wall polysaccharides, including (hemi-) cellulases degrading enzymes (GH10, GH11, GH5, GH6, GH7, GH45, and GH115), pectin degrading enzymes (GH28, GH43, GH51, GH53, GH78, GH88, GH93, PL1, PL3, PL4, and PL9), xylan (GH10, GH11, GH62, GH67, CE1, and CE15), laccase (AA1), cellobiose dehydrogenase (AA3_1), vanillyl alcohol oxidase (AA7), lytic polysaccharide monooxygenases (AA9), and rhamnogalacturonan lyases (PL11) coding genes. Some CAZyme coding genes related to the degradation of non-plant polysaccharides (GH79 and GH88 families) were identified as well in the strain. These data indicate that *A. sydowii* 29R-4-F02 has a strong ability to degrade organic matter to facilitate its growth energy in lignite-containing subseafloor sediments.

**Table 2:** Comparison of CAZyme encoding genes in different species of *Aspergillus*.

Species	GH	GT	PL	CE	CBM	AA
*A. sydowii* 29R-4-F02	337	126	23	39	67	78
*A. parasiticus* CBS 117618	315	127	25	33	51	81
*A. transmontanensis* CBS 130015	309	125	26	32	55	82
*A. arachidicola* CBS 117612	321	132	24	32	54	84
*A. novoparasiticus* CBS 126849	319	133	24	38	52	90
*A. sergii* CBS 130017	321	128	28	33	56	82
*A. flavus* CBS 128202	314	123	25	28	41	69
*A. sojae* CBS 100932	315	127	26	34	52	85
*A. parvisclerotigenus* CBS 121.62	310	128	25	29	49	68
*A. oryzae* RIB 40 FGS	302	119	26	27	41	65
*A. minisclerotigenus* CBS 117635	313	124	26	31	51	74
*A. caelatus* CBS 763.97	250	104	20	26	38	63
*A. pseadocaelatus* CBS 117616	323	132	26	31	49	80
*A. pseudotamarii* CBS 117625	321	125	27	28	51	76
*A. tamarii* CBS 117626	307	131	27	31	45	72
*A. pseudonomius* CBS 1193.88	289	127	24	27	59	75
*A. nomius* IBT 12657	286	126	23	27	50	74
*A. bombycis* CBS 117187	296	122	22	29	52	79
*A. bertholletius* IBT 29228	287	124	20	26	46	69
*A. alliaceus* CBS 536.65	283	131	21	28	41	66
*A. albertensis* IBT 14317	278	135	21	27	46	67
*A. coremiiformis* CBS 553.77	177	109	10	18	29	36
*A. leporis* CBS 151.66	291	127	22	27	49	68
*A. avenaceus* NRRL 4517	241	119	19	27	42	62
*A. steynii* CBS 112812	278	109	24	33	49	64
*A. campestris* CBS 538.81	176	94	10	15	38	36
*A. terreus* NIH2624	277	104	15	28	61	57
*A. nidulans* CBS 126972	272	97	25	28	57	54
*A. fumigatus* Af293CBS126847	264	106	14	27	55	35
*A. niger* ATCC1015	252	121	10	20	44	62

### Gene family evolution and phylogenetic analysis

**Figure 6:**
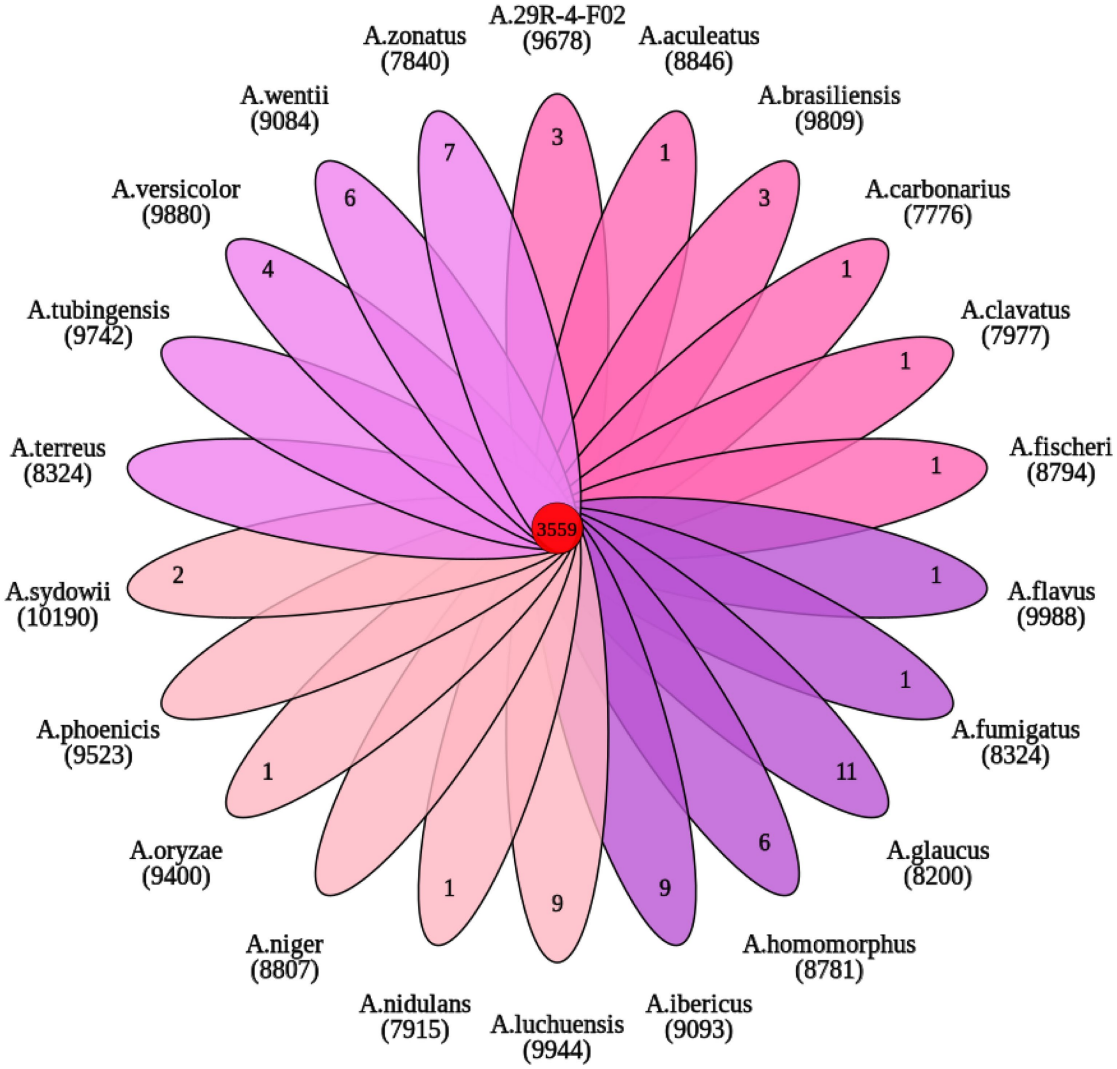
Venn diagram of orthologous gene families.

OrthoFinder analysis showed that 9,678 gene families were identified from 22 sequenced fungi. Among them, 3,559 gene families were shared by 22 fungi and 364 gene families were unique to *A. sydowii* 29R-4-F02 (Figure 6). These 364 gene families are composed of species-specific genes (8) and uncluster genes (356). Of these, the species-specific genes (A00461, A03330, A06713, and A12243) with functional annotation were found to be primarily associated with vesicle fusion and autophagy. 185 uncluster genes (Supplementary Figure 3), as determined through gene functional annotation, were found to be mainly involved in biological processes, cell components and molecular functions according to the results of GO enrichment analysis (Supplementary Figure 4; Supplementary Table 5). These data reveal that *A. sydowii* 29R-4-F02 has the ability to adapt to complex subseafloor environments.

**Figure 7:**
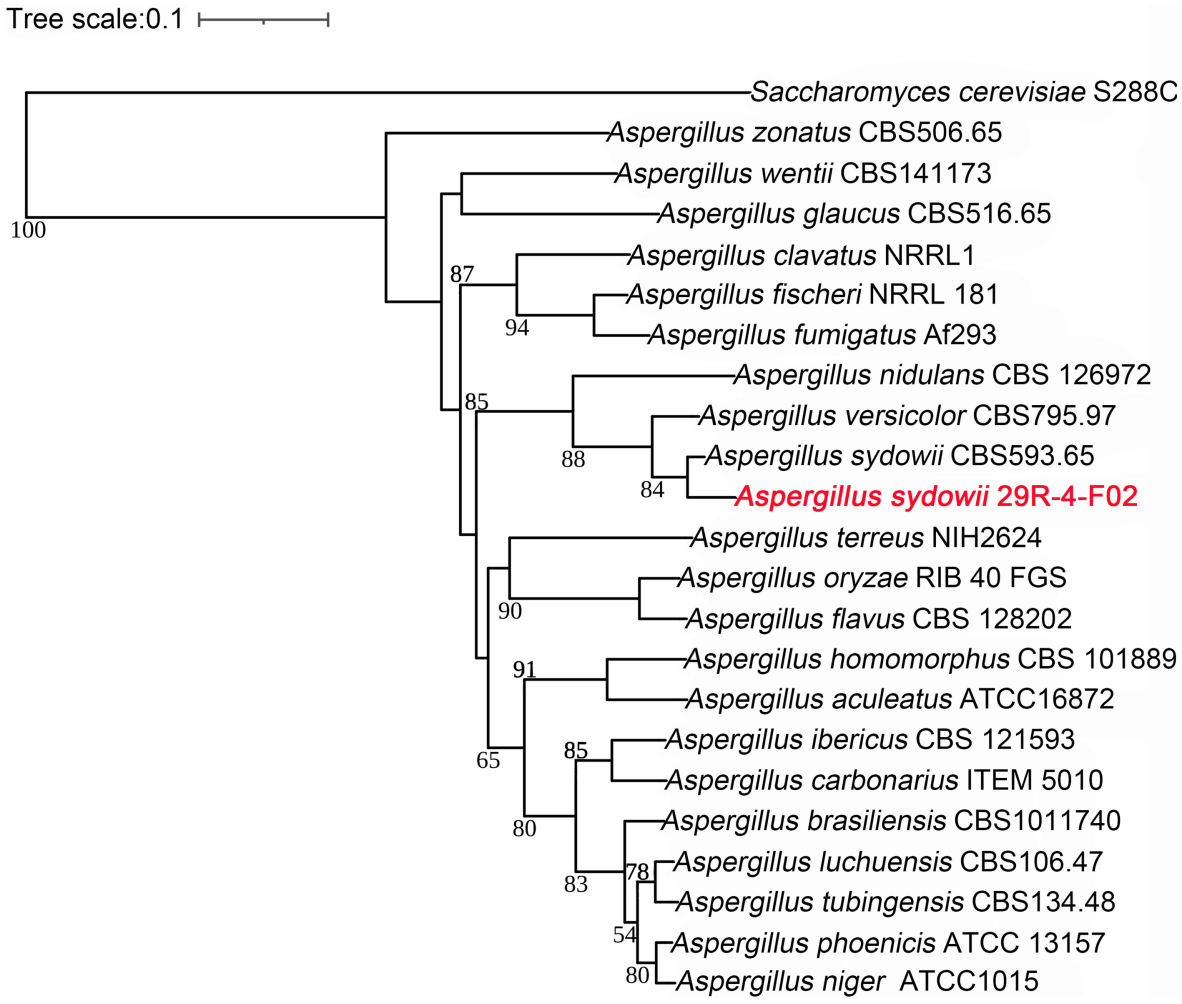
Phylogenetic tree of 21 *Aspergillus* genomes constructed based on single copy ortholog genes. The newly sequenced species in this study are represented in red font. *Saccharomyces cerevisiae* S288C as an outgroup species, the scale bar indicates evolutionary distance. Bootstrap values of <50 have been removed for clarity.

A phylogenetic tree was constructed based on 386 single copy orthologous genes identified from 22 fungi. The inference shows that *A. sydowii* 29R-4-F02 and *A. sydowii* CBS593.65 were clustered into the same clade (Figure 7). Compared with strain CBS593.65, strain 29R-4-F02 had 1,168 unique genes, including 12 genes belonging to 2 species-specific families (OG0000029 and OG0000322) and 1,156 uncluster genes (Supplementary Figures 5, 6; Supplementary Table 6). Among them, OG0000029 and OG0000322 were involved in ZEB2-regulated ABC transporter and glycopeptide α-N-acetylgalactosaminidase, respectively. ZEB2 regulated ABC transporters have been shown to play a crucial role in secreting virulence factors or compounds in pathogenic fungi (Lee et al., 2011; Zhang et al., 2023). Glycopeptide α-N-acetylgalactosaminidase belongs to glycoside hydrolase family (GH101) and has been primarily demonstrated to hydrolyze core 1-type O-glycans from glycoproteins (Fujita et al., 2005). Although this enzyme has been reported in bacteria, its presence in fungi has not been documented yet. In addition, 36 gene families involved in energy metabolism and environmental adaptation were significantly expanded in the genome of *A. sydowii* 29R-4-F02 (Supplementary Table 7). For example, OG0000024, OG0000315, OG0000220, and OG0000464 were associated with NADP-dependent alcohol dehydrogenase, short-chain dehydrogenase, heat shock protein, and nucleotide-excision repair, respectively. Given the nucleotide-excision repair mechanism has been proven to play an important role in genome conservation among subsurface microbes (Becraft et al., 2021; Liu et al., 2022), we believe that these extended genes may provide the genetic basis necessary for the long-term survival of *A. sydowii* 29R-4-F02 in extreme subseafloor sedimentary environments.

### Positive selection improves the environmental adaptation of *A. sydowii* 29R-4-F02

Positive selection, acting on the evolution of functionally important gene families, is an important driving force for microorganisms in adaptation to the complex environment. To investigate those genes related to the adaptability of *Aspergillus* to the subseafloor environment, we conducted a positive selection analysis between *A. sydowii* 29R-4-F02 and *A. sydowii* CBS593.65 using KaKs_Calculator (Ka/Ks > 1 referred to positive selection; Wang et al., 2010). The results showed that 362 genes were under positive selection, including 257 annotated genes and 105 non-annotated genes. Among the annotation genes, 10 genes were involved in CAZymes family, and 5 genes were associated with DNA repair. These genes may help *A. sydowii* 29R-4-F02 to acquire nutrients and energy in lignite containing subseafloor sediments.

## Discussion

*A. sydowii* was a filamentous fungus with a worldwide distribution and attracted our attention due to its abundantly metabolic profile (de Vries et al., 2017; Brandt et al., 2020; Kumar et al., 2021; Jing et al., 2022). In this study, a strain of *A. sydowii* 29R-4-F02 isolated from seafloor sediments was sequenced *de novo* using Nanopore long read sequencing technique. Collinearity and BUSCO analysis showed that strain 29R-4-F02 had high genome quality and integrity. Gene annotation indicated that the strain has rich metabolic activities, especially protein metabolism and energy metabolism, which provided an opportunity for us to explore the lifestyle and ecological adaptation mechanism of fungi in the subseafloor environment.

CAZymes are thought to play an important role in the utilization of organic matter such as cellulose, hemicellulose, pectin and lignin polysaccharides by fungi to obtain nutrients and energy for growth, and are also closely related to host preference and lifestyle adaptation of fungi (Couturier et al., 2012; Ohm et al., 2012; Sista Kameshwar and Qin, 2018). Compared with other species of *Aspergillus*, *A*. *sydowii* 29R-4-F02 had extremely rich CAZymes encoding genes, especially GHs, CEs, and CBMs encoding genes, suggesting that *A*. *sydowii* 29R-4-F02 may have stronger environmental adaptability than other species of *Aspergillus*. In addition, the genome of *A. sydowii* 29R-4-F02 also contains GH79 and GH88 gene families, indicating the ability of this fungus to utilize non-plant polysaccharides, such as animal and bacterial polysaccharides (de Vries et al., 2017; Yu et al., 2020). As more and more sedimentary fungi are obtained (Nagahama et al., 2011; Bengtson et al., 2014; Xu et al., 2014; Burgaud et al., 2015; Drake et al., 2017; Hassett et al., 2019), the analysis of the diversity and abundance of CAZymes encoding genes in these fungi is expected to provide a perspective on how fungi adapt to the extreme subseafloor environment where nutrients are scarce and difficult to utilize.

Based on orthology analysis, we found that *A. sydowii* 29R-4-F02 possesses 364 distinct gene families, among which, the OG0011277 gene family is annotated as a SNARE domain associated with vesicular fusion and autophagy in fungi. Vesicle fusion is the main form of intracellular transmembrane transport of macromolecules and particulate matter (Mohrmann et al., 2010), and autophagy is a strategy for cell renewal of cell components in response to stress or hunger (Glick et al., 2010). In addition, compared with the terrestrial strain *A. sydowii* CBS593.65, *A. sydowii* 29R-4-F02 had more unique genes, including ZEB2-regulated ABC transporters, glycopeptide α-N-acetylgalactoamidosidase, heat shock proteins and nucleotide excision repair genes. ZEB2-regulated ABC transporters are involved in the secretion of virulence factors or compounds of fungi (Coleman and Mylonakis, 2009; Lee et al., 2011), and glycopeptide α-N-acetylgalactosidase is involved in intercellular communication in higher eukaryotes (Fujita et al., 2005). Given that *A. sydowii* 29R-4-F02 was isolated from subseafloor sediment samples at *in situ* temperatures of 50–55°C, such high temperatures may cause damage to DNA, proteins and other biomolecules (Inagaki et al., 2015; Lever et al., 2015). Therefore, strain 29R-4-F02 possesses large number of nucleotides excision, DNA repair, and heat shock protein-related genes, which may be an evolutionary mechanism of heat protection in response to this environment.

## Conclusion

The genome of *A. sydowii* 29R-4-F02 isolated from ~2.4 km of coal-bearing sediments below the seafloor was de vivo sequenced and assembled using a Nanopore long-read sequencing platform. Compared with other species or strains of Aspergillus, *A. sydowii* 29R-4-F02 has more abundant CAZymes encoding genes, which facilitate the fungus to obtain nutrients and energy in the subseafloor sediments. Gene expansion associated with vesicular fusion and autophagy, as well as positive selection of CAZymes and enzymes associated with DNA repair, may be one of the adaptive selection mechanisms that allowed fungi to survive 20 million years in extreme subseafloor environment. The results of this study will help us to understand the life style, evolution and survival mechanism of deep biosphere fungi.

## Data availability statement

The datasets presented in this study can be found in online repositories. The names of the repository/repositories and accession number(s) can be found below: https://www.biosino.org/elmsg/index, LMSG_G000000742.1; https://github.com/liuxuan-425lab/A.sydowii-29R-4-F02.

## Author contributions

J-PJ, XL, and Y-FL performed data analysis. J-PJ wrote the first draft of this manuscript. XL and Y-PZ extracted DNA for genome sequencing. J-PJ and JS cultivated strain of *A. sydowii* 29R-4-F02. C-HL conceived the study and edited the manuscript. All authors contributed to the article and approved the submitted version.

## Funding

This work was supported by the National Natural Science Foundation of China (nos. 41973073, 42273077, and 91951121) and the Science and Technology Innovation Program of Jiangsu Province (no. BK20220036).

## Conflict of interest

The authors declare that the research was conducted in the absence of any commercial or financial relationships that could be construed as a potential conflict of interest.

## Publisher’s note

All claims expressed in this article are solely those of the authors and do not necessarily represent those of their affiliated organizations, or those of the publisher, the editors and the reviewers. Any product that may be evaluated in this article, or claim that may be made by its manufacturer, is not guaranteed or endorsed by the publisher.
