# Supplementary figures and images for: Genomic insights into Aspergillus sydowii 29R-4-F02: unraveling adaptive mechanisms in subseafloor coal-bearing sediment environments
Source: Front Microbiol. 2023 Jun 29;14:1216714. doi: 10.3389/fmicb.2023.1216714 (PMC10339353; doi:10.3389/fmicb.2023.1216714)

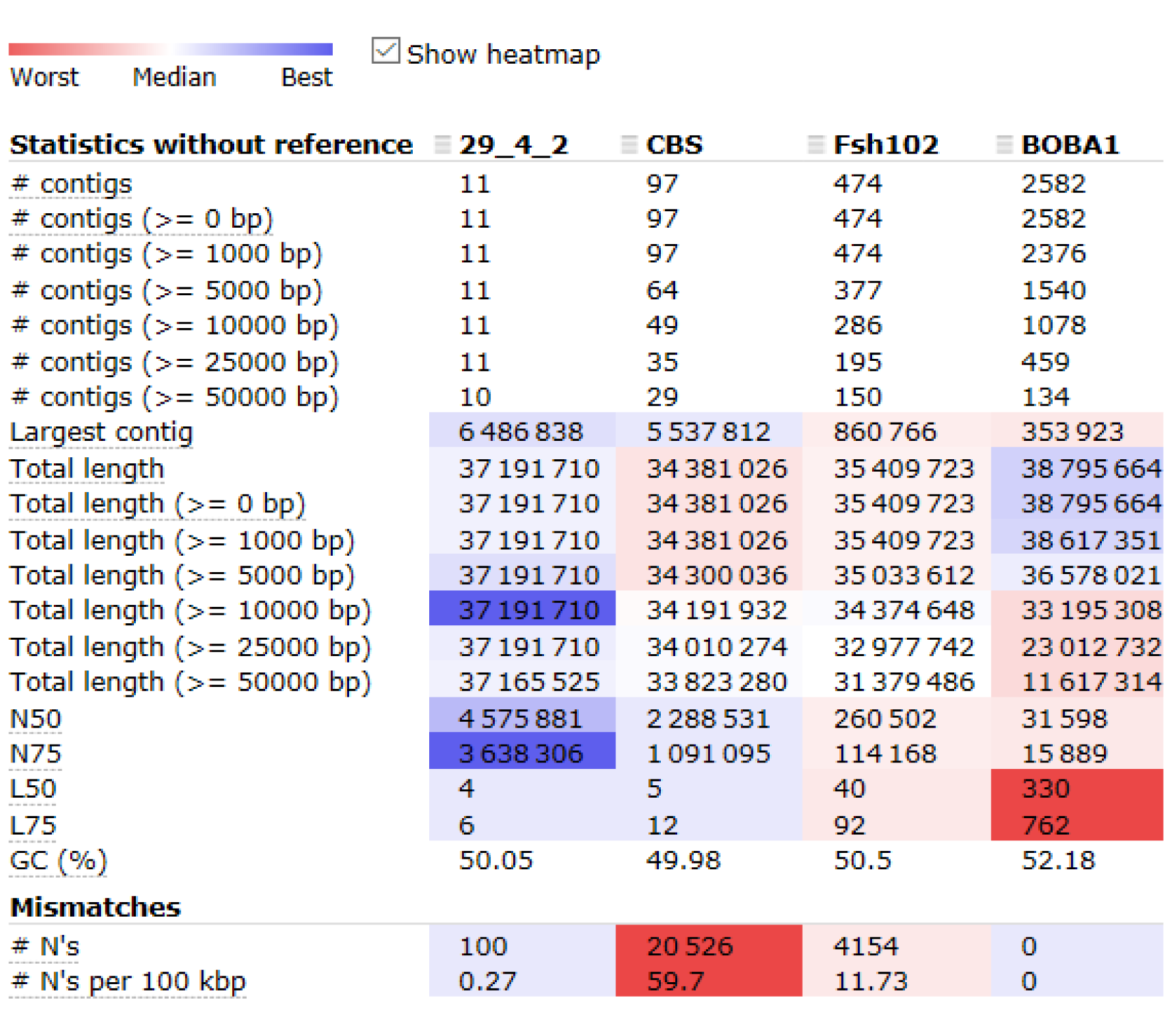

Supplement: Supplementary file 1 [file Data_Sheet_1.zip › Figure S1.TIF]

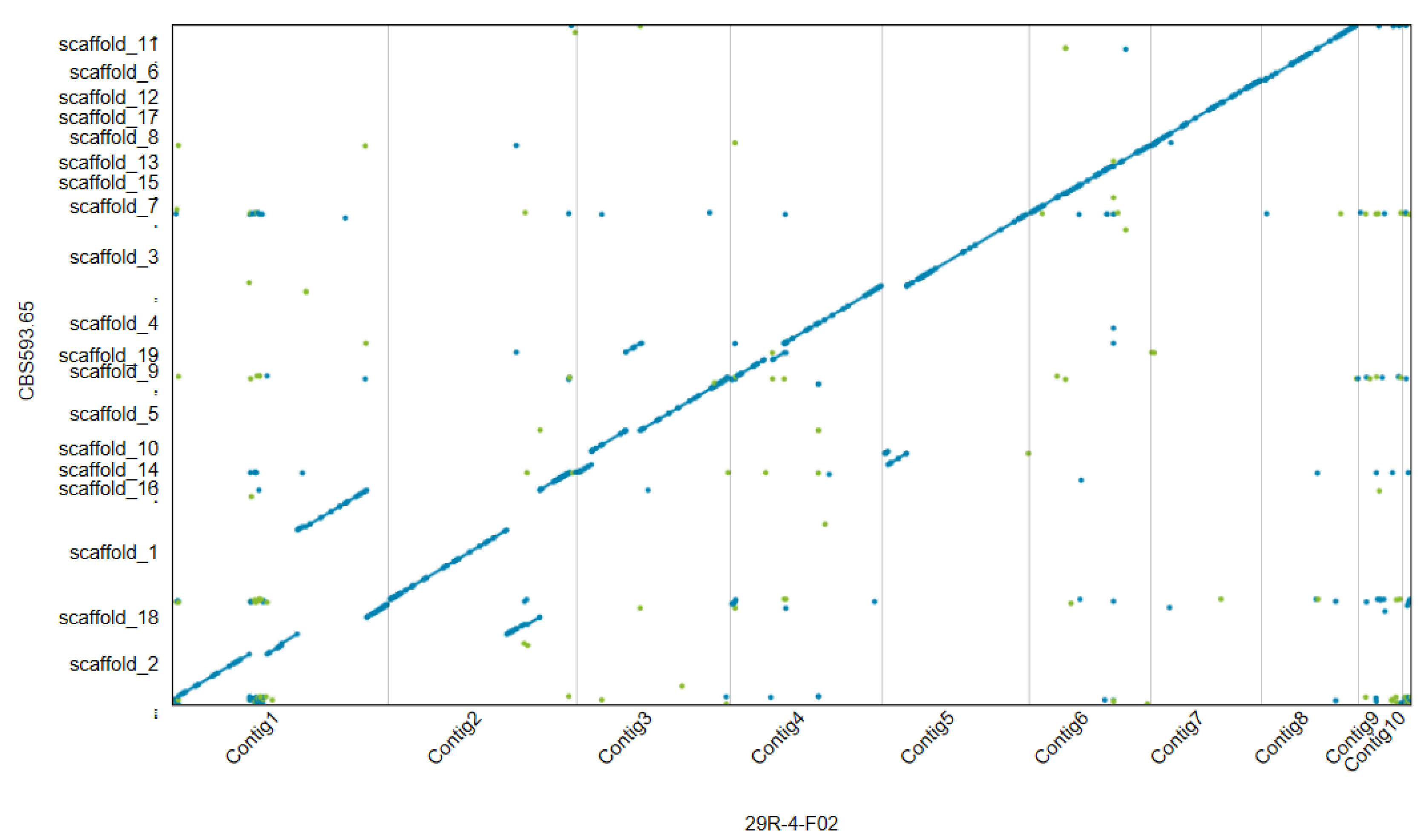

Supplement: Supplementary file 1 [file Data_Sheet_1.zip › Figure S2.TIF]

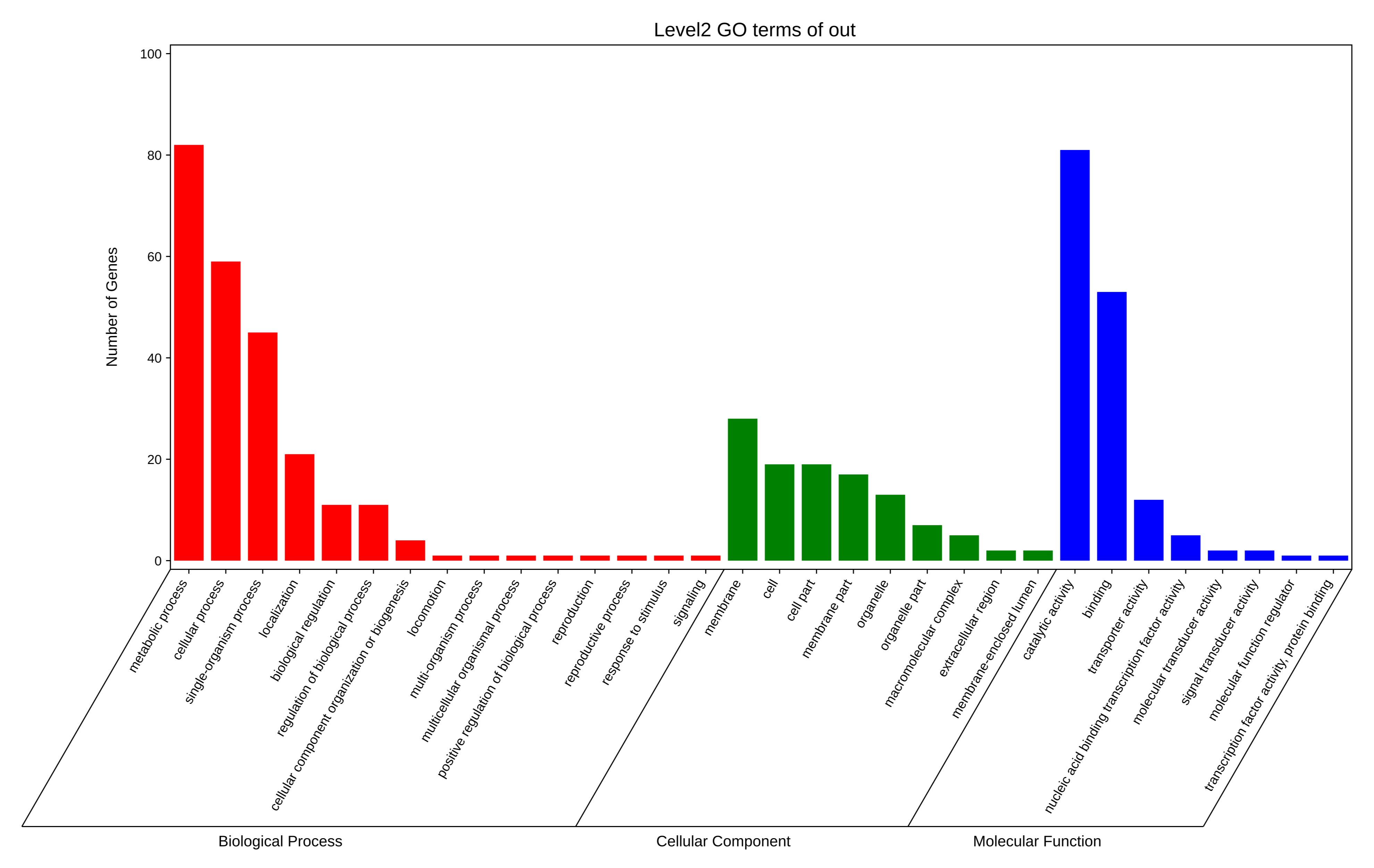

Supplement: Supplementary file 1 [file Data_Sheet_1.zip › Figure S3.TIF]

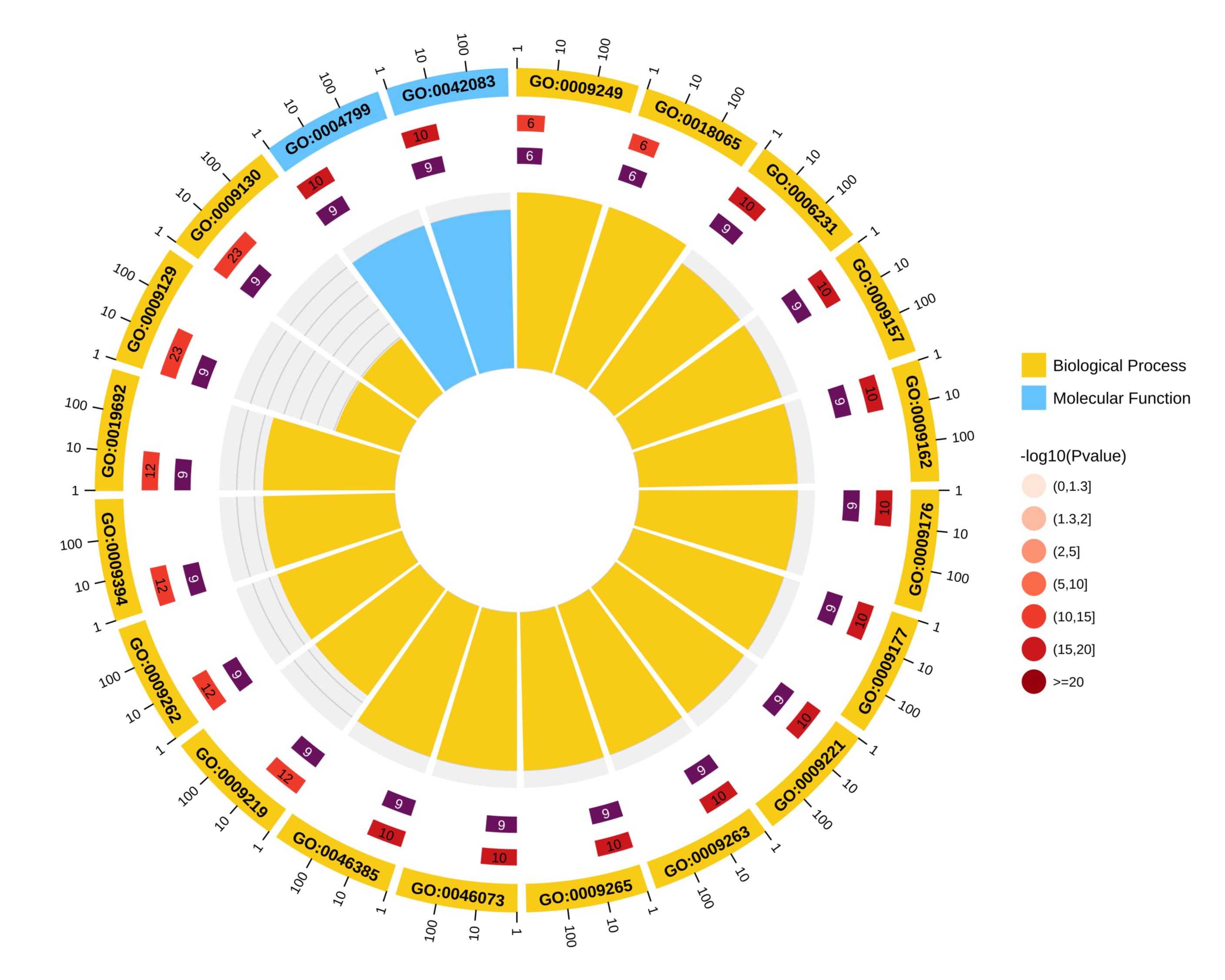

Supplement: Supplementary file 1 [file Data_Sheet_1.zip › Figure S4.TIF]

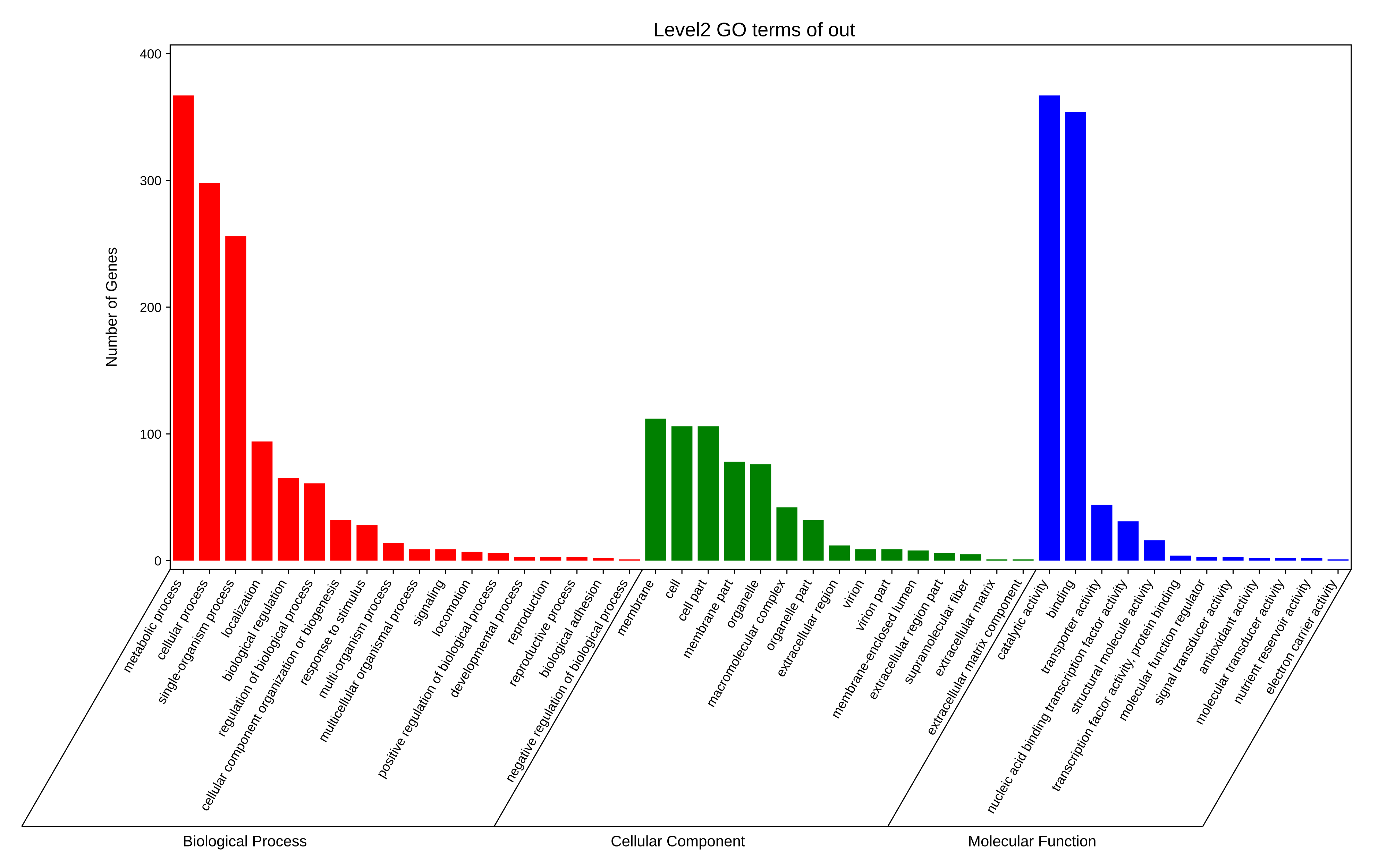

Supplement: Supplementary file 1 [file Data_Sheet_1.zip › Figure S5.TIF]

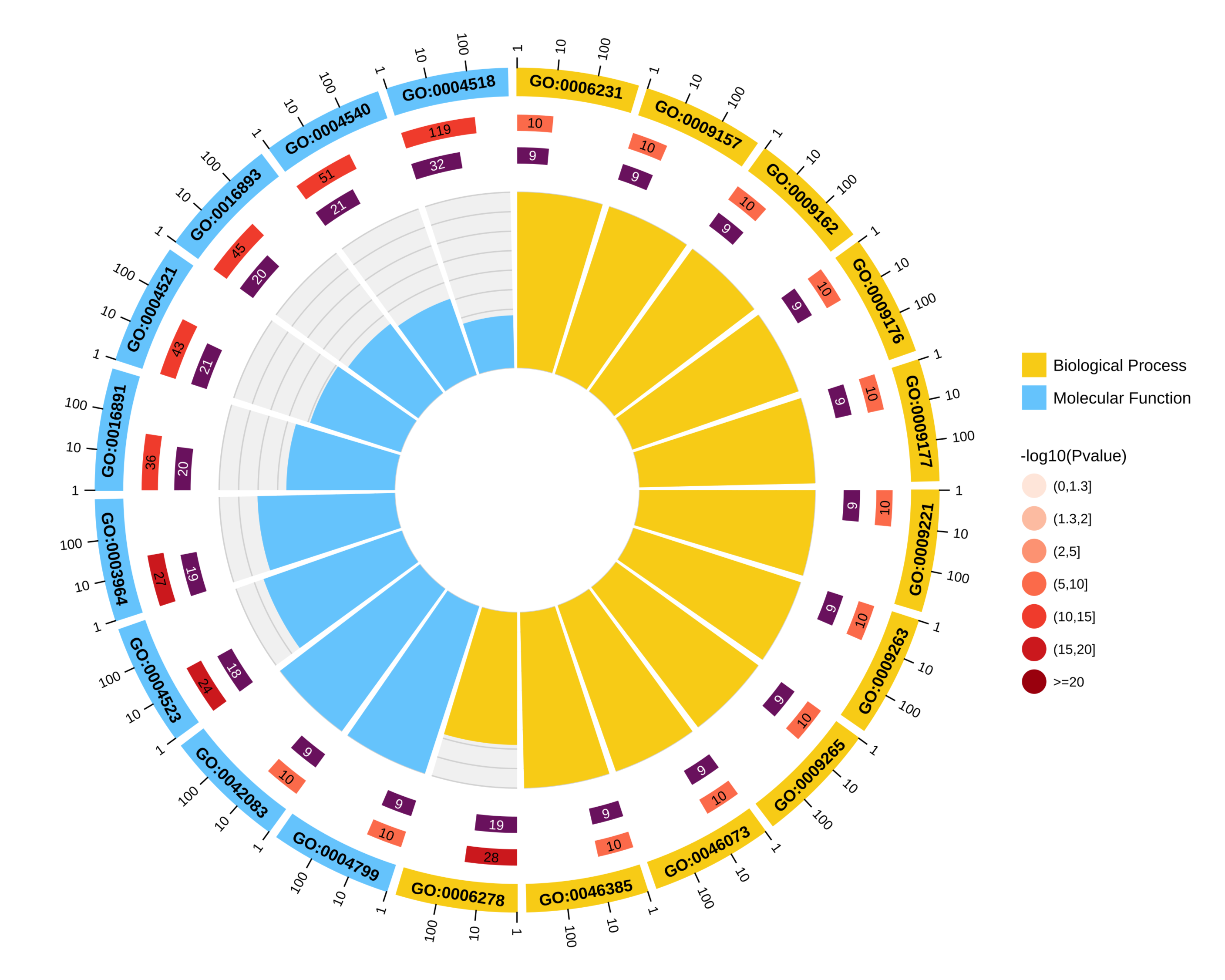

Supplement: Supplementary file 1 [file Data_Sheet_1.zip › Figure S6.TIF]
